# Differentiation of follicular epithelium in polytrophic ovaries of *Pieris napi* (Lepidoptera: Pieridae)—how far to *Drosophila* model

**DOI:** 10.1007/s00709-019-01391-1

**Published:** 2019-05-27

**Authors:** Marta Mazurkiewicz-Kania, Bożena Simiczyjew, Izabela Jędrzejowska

**Affiliations:** Department of Animal Developmental Biology, Institute of Experimental Biology, University of Wrocław, Sienkiewicza 21, 50-335 Wrocław, Poland

**Keywords:** Follicular cells, Cell migration, Intercalation, Lepidoptera

## Abstract

Lepidoptera together with its sister group Trichoptera belongs to the superorder Amphiesmenoptera, which is closely related to the Antliophora, comprising Diptera, Siphonaptera, and Mecoptera. In the lepidopteran *Pieris napi*, a representative of the family Pieridae, the ovaries typical of butterflies are polytrophic and consist of structural ovarian units termed ovarioles. Each ovariole is composed of a terminal filament, germarium, vitellarium, and ovariole stalk. The germarium houses developing germ cell clusters and somatic prefollicular and follicular cells. The significantly elongated vitellarium contains linearly arranged ovarian follicles in successive stages of oogenesis (previtellogenesis, vitellogenesis, and choriogenesis). Each follicle consists of an oocyte and seven nurse cells surrounded by follicular epithelium. During oogenesis, follicular cells diversify into five morphologically and functionally distinct subpopulations: (1) main body follicular cells (mbFC), (2) stretched cells (stFC), (3) posterior terminal cells (pFC), (4) centripetal cells (cpFC), and (5) interfollicular stalk cells (IFS). Centripetal cells are migratorily active and finally form the micropyle. Interfollicular stalk cells derive from mbFC as a result of mbFC intercalation. Differentiation and diversification of follicular cells in *Pieris* significantly differ from those described in *Drosophila* in the number of subpopulations and their origin and function during oogenesis.

## Introduction

Lepidoptera (moths and butterflies) is one of the largest insect orders, including about 160,000 described species. Moreover, hundreds of new lepidopteran species have been described annually in recent years (Kristensen et al. 2007). Despite the species richness of the Lepidoptera, they are far more homogeneous, structurally and ecologically, than the other larger insect groups (Diptera, Coleoptera). Given their popularity associated with their visual attractiveness, the life histories and geographic distributions of Lepidoptera are probably the best known among insects. Lepidopterans provide important model systems for studies of genetics, physiology, development, ecology, and evolutionary biology (Kristensen et al. 2007). Lepidoptera are closely related to Trichoptera, and both form the common group Amphiesmenoptera (Kristensen 1999; Kristensen et al. 2007). The monophyly of this superorder is widely accepted (Kristensen 1999; Kjer et al. 2002; Holzenthal et al. 2006). Amphiesmenoptera are thought to be the sister group of Antliophora, a superorder comprising Diptera, Siphonaptera, and Mecoptera.

The Pieridae is a large family of butterflies with about 76 genera, containing about 1100 species, mostly from tropical Africa and tropical Asia. The European fauna includes about 50 species, of which 17 live in Poland.

Ovary organization and oogenesis are stable characters at the family, and usually order, level. Therefore, data from studies of the ovary structure and oogenesis can be used in phylogenetic considerations, supporting one or the other phylogenetic concept. Follicular epithelium differentiation is one of the characters used in phylogenetic analyses. Previous, detailed comparative investigations of follicular epithelium morphogenesis in the phylogenetic context were carried out in representatives of dipterans—Brachycera and Nematocera (Kubrakiewicz et al. 2003; Mazurkiewicz and Kubrakiewicz 2005, 2008; Tworzydlo et al. 2005; Jaglarz et al. 2008, 2009, 2010).

Among insects, the best investigated group with respect to follicular cell differentiation is flies. However, differentiation of follicular cells has been precisely described only in *Drosophila melanogaster*, one of the most extensively studied model organisms in developmental biology (Ray and Schüpbach 1996; Deng and Bownes 1998; Dobens and Raftery 2000; López-Schier 2003). In *Drosophila,* an initially uniform population of follicular cells progressively and coordinately diversifies into several specialized subgroups (subpopulations) that exhibit different behavior and perform different functions during oogenesis. The basic function of follicular cells in insects is the formation of egg envelopes. Various subpopulations of follicular cells are involved in the formation of different parts of the egg capsule. For instance, in *Drosophila*, a group of follicular cells (border cells) produces a micropyle, a perforated fragment of the eggshell that enables sperm entry (Dobens and Raftery 2000). Moreover, it has been evidenced that spatially restricted inductive interactions between particular follicular cell subsets and the germline cells are responsible for the establishment of the oocyte asymmetry (López-Schier 2003; Steinhauer and Kalderon 2006). During follicular epithelium differentiation in *D. melanogaster*, the migration of some subpopulations of follicular cells has been observed. Some subgroups only change their shape and position within the egg chamber while some of them actively migrate for long distances (for example border cells) (Deng and Bownes 1998; Montell 2003, 2006).

Comparative analyses of the follicular cells’ morphogenesis in the egg chambers of several dipteran species representing main phylogenetic lineages revealed that although the mechanisms of germ- and somatic line cell differentiation recognized in *Drosophila* ovaries are essentially shared by all the dipterans, the representatives of various phylogenetic lineages may exhibit significant differences from the *Drosophila* type (Kubrakiewicz et al. 2003; Mazurkiewicz and Kubrakiewicz 2005; Tworzydlo et al. 2005; Jaglarz et al. 2009, 2010).

Follicular epithelium differentiation in Lepidoptera has been studied in several species: *Hyalophora cecropia* (King and Aggarwal 1972), *Ephestia kuehniella* (Cummings 1972; Cruickshank 1973; Torres 1981), *Bombyx mori* (Yamauchi and Yoshitake a, b) but data from these investigations are fragmentary and concentrate mainly on the contribution of the follicular cells in vitellogenesis and eggshell formation (Kawaguchi et al. 1996, 2000; Sarto et al. 2005; Candan et al. 2008).

In this paper, we provide the first detailed description of the follicular epithelium differentiation and diversification in butterflies.

## Materials and methods

In this paper, we used polytrophic ovaries of *Pieris napi*, a butterfly from the family Pieridae. Females of *Pieris* were collected in SW Poland in the period 2008–2010.

### Preparation of whole mounts

The ovaries were dissected and fixed for 40 min in 4% formaldehyde in phosphate-buffered saline PBS (NaCl, 137 mM; KCl, 2.7 mM; Na_2_HPO_4_, 8 mM; KH_2_PO_4_, 1.5 mM) containing 0.1% Triton X-100. After a few rinses with PBS, the material was first examined with a stereomicroscope Olympus SZX 10 and a light microscope equipped with Nomarski optics and then subjected to whole-mount fluorescent staining. For detection of cell nuclei (DNA), the material was stained with 0.2 mg/ml DAPI (4′,6 diamidino-2′-phenylindole dihydrochloride) (Sigma, D9542) for 20 min in darkness. For detection of microfilaments (F-actin), the ovaries were stained with 2 mg/ml rhodamine-conjugated phalloidin (Sigma, P1951) for 20 min in darkness. In both cases, after rinsing with buffer, the ovarioles were whole-mounted onto microscope slides and examined with either an Olympus BHS light microscope equipped with an epifluorescence device or with an Olympus FV1000 confocal microscope.

### Histological and ultrastructural analysis

Ovaries were dissected and fixed at RT in 2.5% glutaraldehyde in 0.1 M phosphate buffer (pH = 7.4) for a few weeks. The material was rinsed several times with phosphate buffer and postfixed in a mixture containing 1% osmium tetroxide and 0.8% potassium ferrocyanide for 1 h (according to McDonald, 1984). After dehydration in a graded series of acetone, the material was embedded in Epon 812 (Serva, Heidelberg, Germany). Semithin sections (0.6 μm thick) were stained with 1% methylene blue and examined with the Olympus BHS microscope. Ultrathin sections were contrasted with uranyl acetate and lead citrate according to the standard methods and examined with a Zeiss EM 900 electron microscope at 80 kV.

## Results

### Morphology of the ovary

**Fig. 1:**
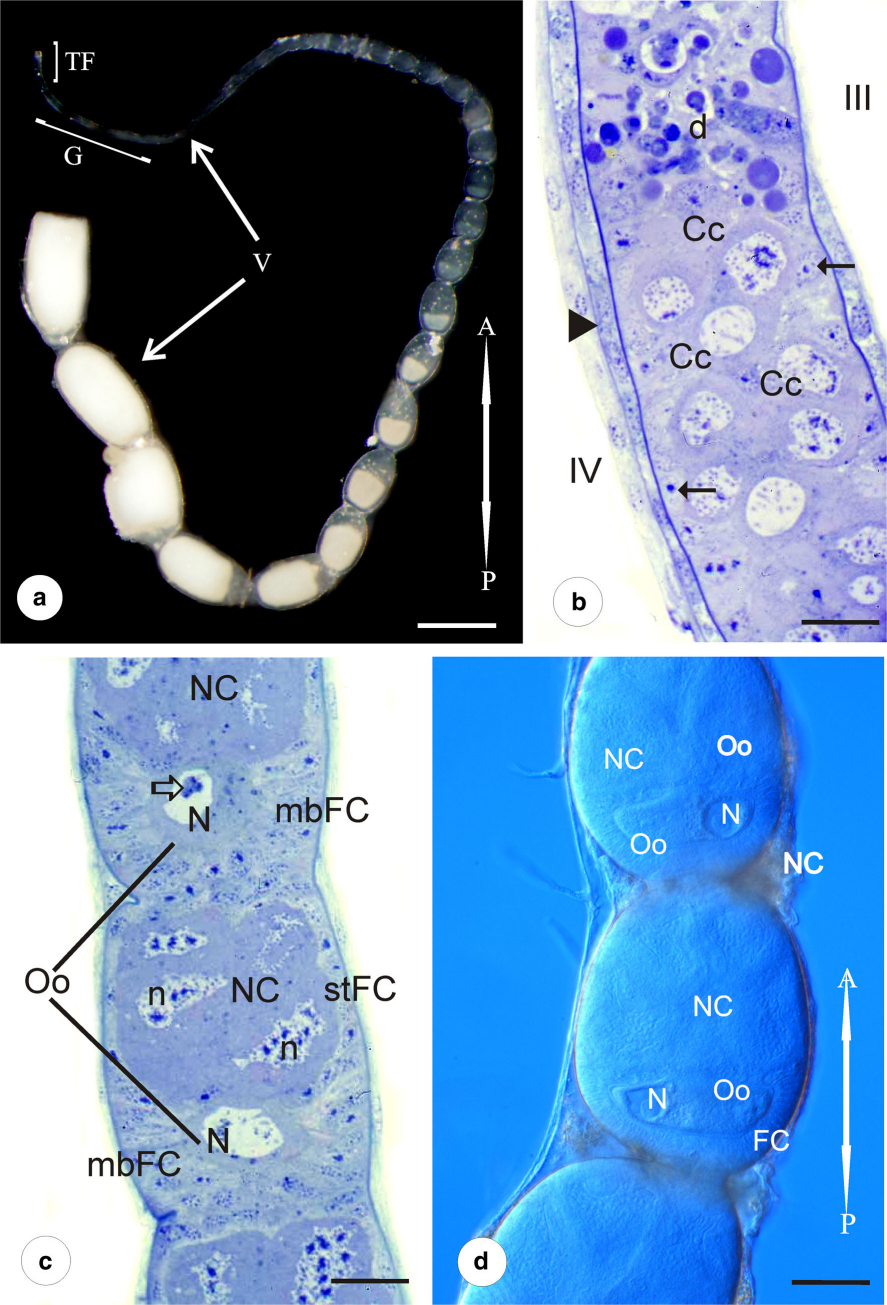
Morphology of the ovariole. **a** The ovariole consists of terminal filament (TF), germarium (G), and vitellarium (V). In vitellarium, numerous egg chambers in consecutive stages of oogenesis are arranged linearly. (A-P) refers to anterior-posterior axis of the ovariole. Stereomicroscope. Whole mount preparation. Scale bar = 1 mm. **b** The part of germarium with zones III and IV. In zone III, degenerating cells (d) are visible. Zone IV is filled with cystocytes in first meiotic prophase (Cc). Arrows indicate prefollicular cells in the peripheral parts of the ovariole. Arrowhead–ovariolar sheath. Semithin section after methylene blue. Scale bar = 40 μm. **c** The part of vitellarium with egg chambers in early previtellogenic stages. Nurse cells (NC) occupy the anterior part of the egg chamber, while the oocyte (Oo) is located in its posterior part. In the nurse cell nuclei (n), patches of dense material are visible. Relatively large oocyte nucleus (N) occupies a central position in the ooplasm. mbFC, mainbody follicular cells; stFC, stretched follicular cells. Hollow arrow indicates nuclear body in the oocyte karyoplasm. Semithin section after methylene blue. Scale bar = 40 μm. **d** The part of vitellarium with egg chambers in advanced previtellogenic stages. Oocyte (Oo) nucleus (N) is visible on either side of the ooplasm. FC, follicular cells; NC, nurse cells. (A-P) refers to anterior-posterior axis of the ovariole. Whole mount preparation, Nomarski optics. Scale bar = 50 μm

**Fig. 2:**
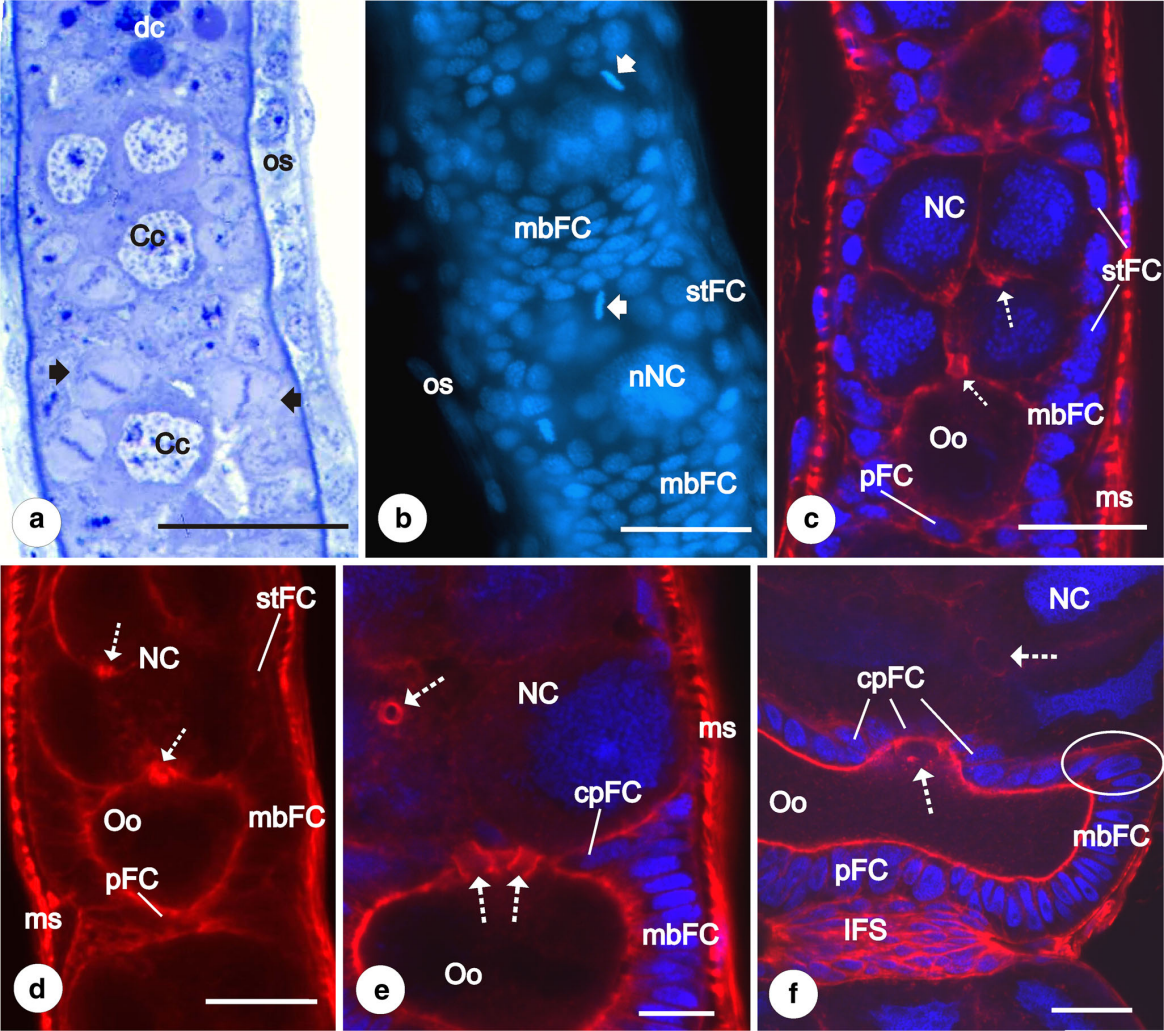
Previtellogenesis. Early stages of follicular epithelium differentiation. **a** Part of germarium:zones III and IV. Clusters of cystocytes (Cc) are surrounded by dividing follicular cells (arrows). In the basal part of zone III, a group of degenerating cells (dc) is visible. os, ovariole sheath. Semithin section after methylene blue. Scale bar = 40 μm. **b** Anterior part of vitellarium after DAPI staining. mbFC, main body follicular cells; nNC, nurse cells nuclei; os, ovariole sheath; stFC, stretched follicular cells. Arrows indicate dividing follicular cells. Fluorescence microscope, whole mount preparation. Scale bar = 50 μm. **c**, **d** Early previtellogenic egg chamber. mbFC, main body follicular cells; ms, muscles covering the ovariolar sheath; NC, nurse cells; Oo, oocyte; pFC, posterior follicular cells; stFC, stretched follicular cells. Cystocytes within the clusters remain interconnected by intercellular bridges (dotted arrows). Confocal microscope, whole mount preparation after DAPI/phalloidin-conjugated rhodamine (**c**), only phalloidin-conjugated rhodamine (**d**). Scale bar = 50 μm. **e** Part of the previtellogenic egg chamber after DAPI/phalloidin-conjugated rhodamine. Third subpopulation of follicular cells is distinguishable—centripetal cells (cpFC). mbFC, main body follicular cells; ms, muscle covering the ovariole sheath; NC, nurse cells. Dotted arrows indicate interfollicular bridges connecting the oocyte (Oo) with nurse cells and nurse cell with nurse cell. Confocal microscope, whole mount preparation, scale bar = 50 μm. **f** Part of the late previtellogenic egg chamber after DAPI/phalloidin-conjugated rhodamine. Centripetal cells (cpFC) surround intercellular bridges, grouped at the apical part of the oocyte (dotted arrow) and connecting the oocyte (Oo) with the nurse cells (NC). mbFC, main body follicular cells; pFC, posterior cells connected directly with the interfollicular stalk cells (IFS). Some centripetal cells still contact the basal lamina and form protrusions toward the anterior pole of the oocyte (encircled). Confocal microscope, whole mount preparation, scale bar = 50 μm

Each of the paired ovaries of *Pieris napi* is composed of four long ovarioles of meroistic polytrophic type (Fig.1a). Individual ovarioles are covered by a relatively thick ovariolar sheath and a layer of muscles (Fig. 2a, c–e). Each ovariole is built of four linearly arranged parts: terminal filament, germarium, vitellarium, and ovariolar stalk. Terminal filaments join up with each other and form a ligament that attaches the gonad to the body wall.

**Fig. 3:**
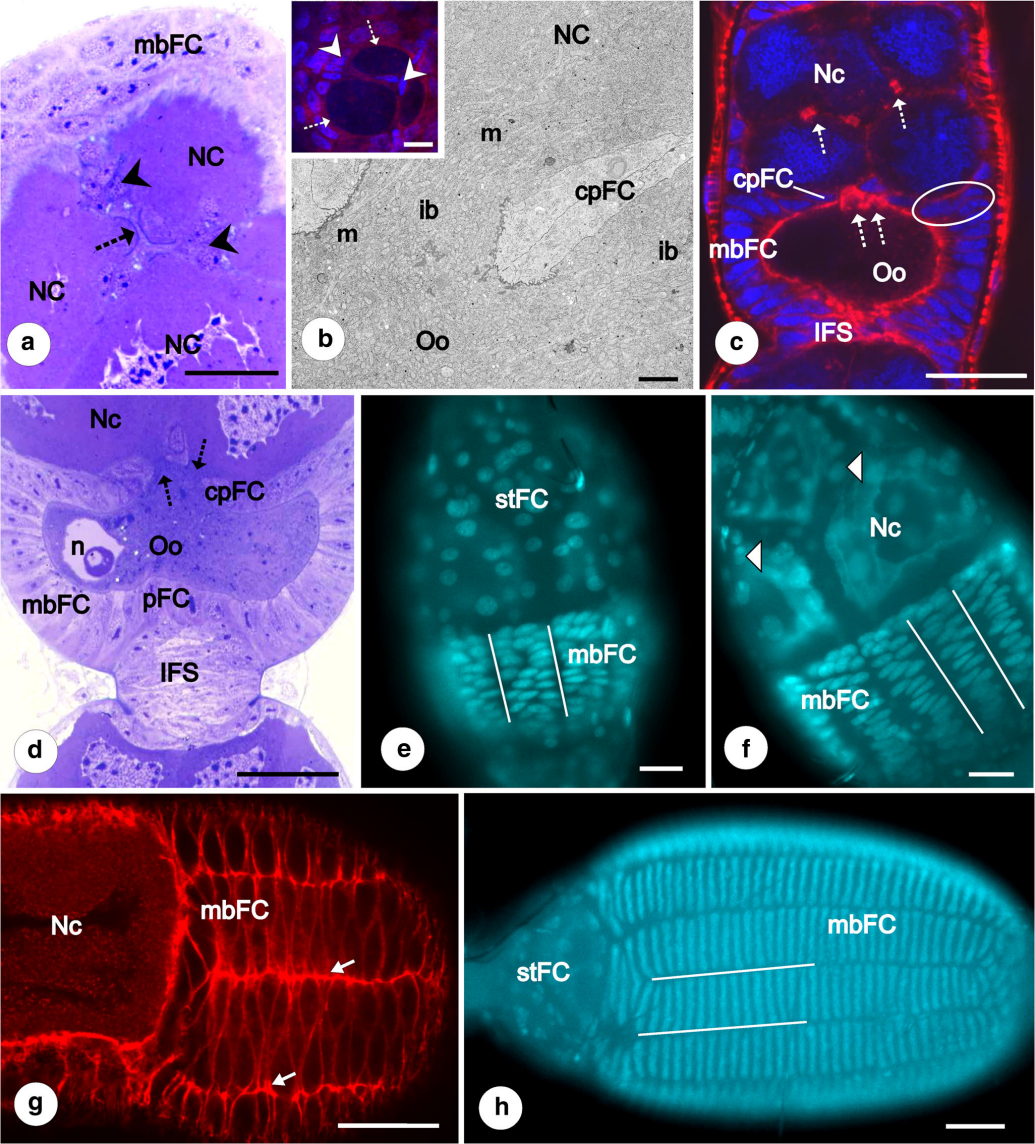
Previtellogenesis and vitellogenesis. Subpopulations of follicular epithelium. **a–c** Cross section of previtellogenic egg chamber in the plane of the intercellular bridges (dotted arrows on A,C; ib. on B) that connect the nurse cells (NC) with oocyte (Oo). Within the intercellular bridge mitochondria (m) and ribosomes are visible. mbFC, main body follicular cells. Around the bridges, centripetal cells (cpFC on B, C; arrowheads on A) without contact with the basal lamina are visible. **a** Semithin section after methylene blue. Scale bar = 40 μm. **b** TEM. Scale bar = 1.7 μm. Insert DAPI//phalloidin-conjugated rhodamine. Confocal microscope, whole mount preparation. Scale bar = 50 μm; **c** DAPI//phalloidin-conjugated rhodamine. Interfollicular stalk (IFS) becomes visible. Centripetal cells with contact of basal lamina are encircled. Confocal microscope, whole mount preparation. Scale bar = 50 μm. **d** Part of the late previtellogenic egg chamber. Interfollicular stalk (IFS) becomes more prominent. Dotted arrows indicate intercellular bridges connecting the nurse cells with the oocyte. cpFC, centripetal cells; mbFC, main body cells; NC, nurse cells; n, oocyte (Oo) nucleus; pFC, posterior cells in direct contact with the interfollicular stalk cells. Semithin section after methylene blue. Scale bar = 40 μm. **e–h** Intercalation of main body follicular cells (mbFC). The process of intercalation started from the posterior pole of the oocyte (**e**). Firstly, the rows (selected by straight lines) are two or three cells wide (**e**, **f**). The borders between the rows are clearly marked by F-actin rich cellular projections (arrows) (**g**). Finally, mbFC are arranged into regular single cell rows (**h**). NC, nurse cells; mbFC, main body follicular cells; stFC on (**e**) and arrowhead on (**f**) stretched follicular cells, (A-P) refers to anterior-posterior axis of the ovariole. **e**, **f**, **h** Fluorescence microscope whole mount preparation after DAPI staining, scale bar = 40 μm; **g** Confocal microscope, whole mount preparation after phalloidin-conjugated rhodamine, scale bar = 50 μm

The thin and elongated germarium houses germ cells and prefollicular and follicular tissues. The germarium is divided into four zones. Zone I is the most apical part of the germarium, in which the germ cells differentiate into cystoblasts and divide mitotically. Zone II is the region in which the undifferentiated germ cell clusters (groups of interconnected cystocytes) arise (not shown). Zone III, called a “control zone,” is filled with numerous cells that undergo degeneration (apoptotic cells) (usually whole germ cell cluster) (Figs.1b and 2a). In zone IV, the distalmost region of the germarium, the germ cell clusters are fully formed (Fig. 2a). The cystocytes of the cluster are interconnected by cytoplasmic bridges (ring canals) and arranged like the elements of a rosette. All of them enter the first meiotic prophase (Fig. 1b) as indicates the presence of synaptonemal complexes (not shown).

The significantly elongated, posteriorly located vitellarium constitutes most of the ovariole length. It is composed of tens of linearly arranged egg chambers in progressively advanced stages of oogenesis. Each egg chamber contains an eight-germ-cell cluster covered by the somatic follicular epithelium. Cystocytes within the cluster are diversified into one posteriorly located oocyte and seven nurse cells (trophocytes) that occupy anterior part of the egg chamber. The oocyte maintains connection with three directly neighboring nurse cells by intercellular bridges that are located on the apical part of the oocyte (Figs. 2c–f, and 3a, c, d).

The volume of oocyte increases in two consecutive stages of growth: previtellogenesis and vitellogenesis. During previtellogenesis, various RNA fractions (rRNA, mRNA) and increasing in number organelles are deposited. In vitellogenesis, the oocyte accumulates reserve materials and its volume significantly grows.

Individual egg chambers are separated by interfollicular stalks composed of flattened cells. The ovariolar stalks form the posterior end of the ovarioles. They are composed of groups of somatic cells that link individual ovarioles to the lateral oviduct (not shown).

### Follicular epithelium

Follicular cells differentiate from prefollicular cells located in the germarium, at the border of zones III and IV and close to the wall of the ovariole. Each germ cell cluster comes to be encased by a simple follicular epithelium in which intensely dividing follicular cells have been observed (Fig. 2a). As a result, completely formed egg chambers appear in the most posterior region of zone IV. Less numerous dividing follicular cells have been observed in the vitellarium during previtellogenic growth of the oocyte (Fig. 2b). When the germ cells of the clusters start to differentiate into the oocyte and nurse cells, the initially uniform population of the follicular cells becomes progressively diversified into specialized subgroups. The process of follicular cell diversification occurs in several steps, and finally, in the advanced vitellogenic egg chambers, five distinct subpopulations of follicular cells are distinguishable: (1) main body follicular cells (mbFC) — covering the lateral aspects of the oocyte, (2) stretched follicular cells (stFC) — surrounding the nurse cells compartment, (3) posterior terminal cells (pFC) — located at the posterior pole of the oocyte, (4) centripetal follicular cells (cpFC) — placed between the nurse cells and the oocyte, and (5) interfollicular stalk cells (IFS) — forming the interfollicular stalks (Figs. 5 and 6).

### Early previtellogenesis

**Fig. 4:**
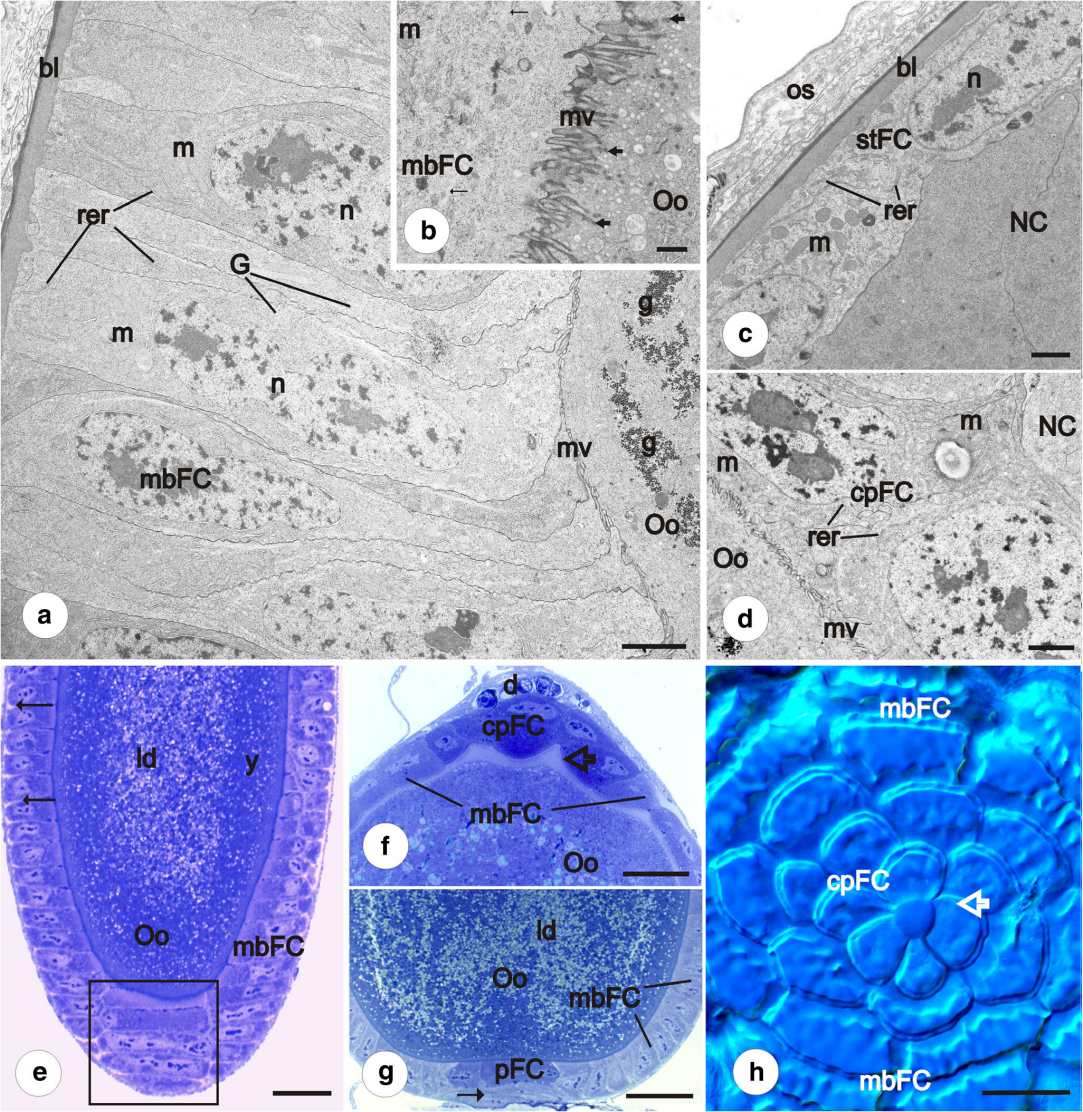
Vitellogenesis and choriogenesis. Subpopulations of follicular epithelium. **a**, **b** Main body follicular cells in early (**a**) and late (**b**) previtellogenesis. TEM. **a** Follicular cells are high. Between oocyte (Oo) and follicular cells (mbFC), poorly developed microvilli (mv) were observed. m, mitochondria; rer, rough endoplasmic reticulum; n, follicular cell nucleus; g, glycogen in the ooplasm; G, Golgi apparatus; bl, basal lamina. **b** Well-developed microvilli (mv) between oocyte (Oo) and follicular cells (mbFC). m, mitochondria; thick arrows indicate endocytic vesicles in ooplasm; thin arrows show microtubule bundles. Scale bar **a** = 2.5 μm, **b** = 1.1 μm. **c** Stretched follicular cells (stFC) based on basal lamina (bl). In the cytoplasm of follicular cells, few organelles were observed. NC, nurse cell; n, follicular cell nucleus; m, mitochondria; os, ovariole sheath; rer, rough endoplasmic reticulum. TEM. Scale bar = 1.1 μm. **d** Contact centripetal follicular cells (cpFC) with the oocyte (Oo). Zone of microvilli (mv) is visible. m, mitochondria; NC, nurse cell; rer, rough endoplasmic reticulum. TEM. Scale bar = 1.7 μm. **e** Advanced vitellogenesis. Between the main body follicular cells (mbFC), spaces are visible (arrows) (epithelial patency). Square shows high and narrow follicular cells after intercalation, arranged in single rows. ld, lipid droplets; Oo, oocyte; y, yolk. Semithin section after methylene blue. Scale bar = 50 μm. **f**, **g**. Advanced vitellogenesis. Centripetal (cpFC) (**f**) and posterior cells (pFC) (**g**) are stained differently from main body follicular cells (mbFC). d, degenerating nurse cells; ld, lipid droplets; Oo, oocyte; y, yolk. The chorion of micropylar apparatus is marked with a hollow arrow. Semithin section after methylene blue. Scale bar = 50 μm. **h** Choriogenesis. Centripetal cells (cpFC) form a characteristic rosette pattern. The main body follicular cells (mbFC) are arranged in rows. Hollow arrow indicates central part of a rosette, in the future —micropylar plate. Whole mount preparation, Nomarski optics. Scale bar = 20 μm

In the newly formed egg chambers, each group of germ cells is covered by a simple follicular epithelium. The volume of the germ cells is similar but the morphology of their nuclei is strikingly different. The oocyte continues the meiotic division, whereas the trophocytes withdraw from the meiosis. The nuclei of trophocytes are large and irregular in shape. They are highly polyploid (Fig. 3f) and contain patches of dense material that intensely stain with methylene blue (Figs. 1c). In the perinuclear region, nuage material is observed (not shown) and whole cytoplasm contains a few mitochondria and ribosomes (Fig. 4c). The relatively large oocyte nucleus (germinal vesicle) occupies a central position in the ooplasm. In the karyoplasm, polymorphic nuclear bodies (Fig. 1c) and meiotic chromosomes (not shown) are observed.

Analysis of the whole mounts in the confocal microscope after DAPI and rhodamine-conjugated phalloidin staining showed that at this stage of oogenesis, two subpopulations of the follicular cells are discernable: the main body follicular cells and the stretched cells (Fig. 2b, c). The main body follicular cells (mbFC) are high and columnar, and closely adjacent to each other. The stretched cells (stFC) are slightly lower and wider (cuboidal) in comparison to the mbFC (Fig. 2c). During previtellogenesis, the activity and volume of the trophocytes increase, and in their cytoplasm numerous organelles (mostly mitochondria and ribosomes) accumulate. As a result, the stretched cells become more flattened (Fig. 2d). In the cytoplasm of both follicle cell subpopulations, a few mitochondria and cisterns of endoplasmic reticulum are visible (Fig 4a, c). In the progress of early previtellogenic growth, some of the main body follicular cells form projections that surround the posterior pole of the oocyte. In consequence, the posterior pole of the oocyte becomes ensheathted by the third subpopulation of the follicular cells termed the posterior terminal cells (pFC) (Fig. 2c, d). The number of follicular cells associated with the posterior pole of the oocyte increases as a result of mitotic divisions.

### Advanced previtellogenesis

**Fig. 5:**
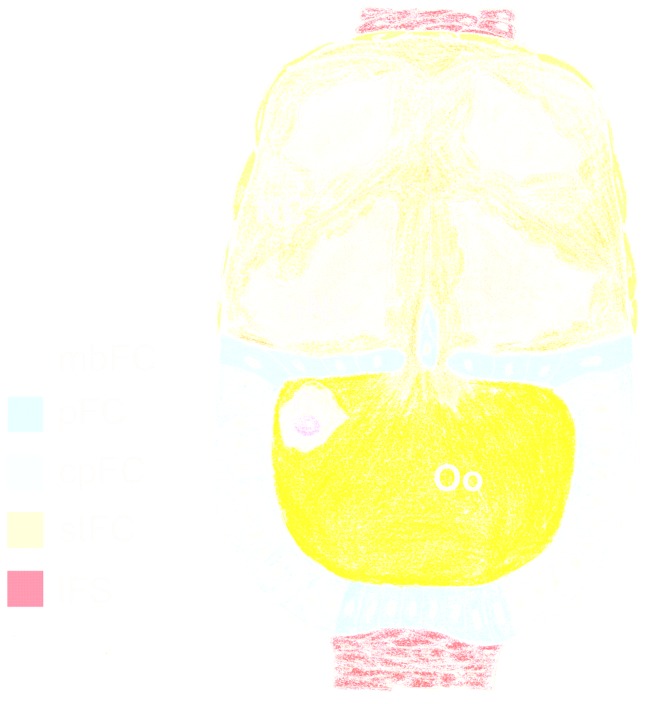
Diagram of early vitellogenic egg chamber of *Pieris napi. NC - nurse cells, nNc - nurse cells nuclei, n - oocyte nucleus, Oo - oocyte  *

As oogenesis progresses, the volume of the oocyte gradually increases. It is partially due to the continuous transfer of organelles (mostly mitochondria and ribosomes) from trophocytes via cytoplasmic bridges to the ooplasm (Fig. 3b). The position of the oocyte nucleus changes from the central to lateral cytoplasm (Figs. 1d, 3d, and 5). Moreover, in advanced previtellogenic oocytes, the germinal vesicle is visible at either side of the ooplasm. It suggests that its position within ooplasm is not fixed. In advanced previtellogenesis, the mbFC occasionally divide, and so their number slightly rises. Some of the mbFC located close to the nurse cells start to migrate between the oocyte and the nurse cell compartments, giving rise to the next FC subpopulation termed centripetal cells (cpFC) (Figs. 2e, f and 3a–d). Most of the centripetal cells during their movement detach from the basal lamina and reach the intercellular bridges grouped at the apical part of the oocyte (Figs. 2f and 3a–d). Those cells surround intercellular bridges connecting the oocyte and the nurse cells in a rosette-like pattern (Fig. 2f). Some of the centripetal cells, located exactly at the border of nurse cells and the oocyte compartments, still contact the basal lamina and form protrusions toward the anterior pole of the oocyte (Figs. 2f and 3a).

During previtellogenic growth, initially uniformly distributed, the mainbody follicular cells gradually intercalate, forming roughly regular rows aligned along the anterior posterior axis of the oocyte (Fig. 7). The process of intercalation starts from the posterior pole of the oocyte (Fig. 3e, f, h). Firstly, the rows are two or three cells wide (Fig. 3e, f). The borders between the rows are clearly marked by F-actin rich cellular projections (Fig. 3g).

With the progress of previtellogenesis, neighboring egg chambers become separated by the interfollicular stalk cells (IFS) (Figs. 2f and 3a, d). Initially, these cells are arranged irregularly, and the stalks take the whole width of the ovariole (Fig. 3a). During later stages, when the number of the interfollicular stalk cells significantly increases, the interfollicular stalks noticeably elongate and the arrangement of stalk cells changes from irregular to radial (Fig. 3d). Some of the interfollicular stalk cells directly adhere to the pFC and stFC, and thus, the follicular cells surrounding the anterior and posterior poles of the egg chambers have no contact with the basal lamina (Figs. 2f and 3d).

### Vitellogenesis

In vitellogenesis, the volume of the oocyte significantly grows since the ooplasm becomes filled with yolk spheres and lipid droplets. The mbFC cease mitotic activity, gradually increase in size, and become more columnar. At that stage of oogenesis, numerous microvilli appear between the oocyte and mbFC (Figs. 4a, b). The microvilli, but at lower number, were also found between the oocyte and the centripetal cells (Fig. 4d), but not between the stretched cells and nurse cells (Fig. 4c). The cytoplasm of the main body follicular cells is filled with a great number of mitochondria, cisterns of endoplasmic reticulum, and Golgi complexes. In cortical ooplasm, many vesicles with electron dense material and microtubules have been observed (Fig. 4b). The cytoplasm of centripetal cells contains accumulations of mitochondria and elements of rough endoplasmic reticulum (Fig. 4d). In turn, in the stretched cells’ cytoplasm, the number of organelles is remarkably lower (Fig. 4c).

During vitellogenesis, the mbFC arranged into roughly regular rows continue intercalation. Intercalating cells are characteristically drop-shaped (Fig. 3g) and redistributed in the posterior-anterior direction. Eventually, they become conspicuously narrow, long and high, arranged into regular one cell thick rows (Fig. 3h). During progressive growth of the oocyte and planar movement of the mbFCs, the interfollicular stalks become more prominent (Fig. 3d). The number of follicular cells forming the stalks significantly increases although they remain mitotically inactive.

In a more advanced vitellogenic stage of egg chamber development, intercellular spaces between the mbFC appear. In consequence, initially coherent epithelium becomes patent (Fig. 4e). The stFC remain squamous and tightly apposed to each other. In the late stages of vitellogenesis, the nurse cells with accompanying follicular stretched cells break down (Fig. 4f). The ooplasm of late vitellogenic oocytes is exceptionally poor in reserve materials (Fig. 4e, g). At this stage, the cytoplasm of centripetal and posterior terminal cells stains more intensely after methylene blue. The centripetal cells adhere directly to the degenerating nurse cell compartment while the posterior terminal cells contact the interfollicular stalk cells (Fig. 4f, g). After degeneration of the nurse cells and their surrounding stretched follicular cells, intercellular bridges become closed. Those centripetal cells that directly reached the intercellular bridges contact each other with their apical membranes on the anterior pole of the oocyte (Fig. 4h). During choriogenesis, these cells will be involved in micropyle formation (Fig. 4f).

## Discussion

### Differentiation of follicular cells in butterflies

As mentioned in the “Introduction” section, data concerning follicular cell differentiation in butterflies are scarce and concentrate largely on the participation of the follicular cells in vitellogenesis and choriogenesis (Swevers and Iatrou 2003; Telfer 2009). Previous investigations indicate that in Lepidoptera, follicular epithelium diversifies into several different subpopulations: cells covering the oocyte, cells surrounding the nurse cells, and cells migrating between the nurse cells and oocyte compartments (centripetal cells) (Yamauchi and Yoshitake 1984b; Santos and Gregório 2006). Additionally, in *Bombyx mori*, three different types of centripetal cells have been distinguished: MRFC (micropylar rosette-forming cells), MCFC (micropylar channel-forming cells), and MOFC (micropylar orifice-forming cells) (Yamauchi and Yoshitake 1984a). Our results have shown that in *Pieris*, follicular epithelium diversifies into five distinct subpopulations: mbFC, stretched cells, posterior terminal cells, centripetal cells, and interfollicular stalk cells. To the best of our knowledge, this is the first detailed description of follicular epithelium differentiation and diversification in butterflies.

Some previously conducted studies focused on early stages of follicular epithelium differentiation in lepidopterans. Santos and Gregório (2006) have shown that in *Diatraea saccharalis* differentiation of follicular epithelium begins in the basal part of the germarium (the distalmost part of zone IV) in early stages of previtellogenesis. A similar situation has been observed in *Bombyx mori* (Yamauchi and Yoshitake 1984b). Thus, the present results confirm that zone IV of the germarium is the place where the follicular epithelium starts to arrange (differentiates). However, in *Pieris*, this process begins before the oocytes enter previtellogenic growth. Aforementioned data indicate that in lepidopterans, as a rule, the follicular epithelium formation takes place in the distal regions of the germarium either just before or at the beginning of previtellogenesis.

Former research carried out on butterflies has shown that the initial step of follicular epithelium diversification leads to the formation of two subpopulations of follicular cells: cells covering the oocyte and cells surrounding the nurse cells (Yamauchi and Yoshitake 1984b; Santos and Gregório 2006). In this study, we revealed that in *Pieris*, follicular epithelium diversification proceeds in four consecutive steps. The first to appear are the mainbody follicular cells and stretched cells, counterparts to the abovementioned cells covering the oocyte and surrounding the nurse cells, respectively. In the next three steps, the posterior terminal cells, centripetal cells, and interfollicular stalk cells differentiate.

Detailed and more or less complete data on the follicular cell differentiation in polytrophic ovaries come from studies on dipterans, neuropterans, and dermapterans (Kubrakiewicz et al. 2003; Mazurkiewicz and Kubrakiewicz 2005, 2008; Tworzydlo et al. 2005, 2010; Tworzydlo and Kisiel 2011; Garbiec and Kubrakiewicz 2012; Mazurkiewicz-Kania et al. 2012). In each of the abovementioned groups, the pattern of follicular epithelium differentiation shows major or minor discrepancies. The best investigated with respect to follicular cell differentiation are flies, including *Drosophila.* Since butterflies are more closely related to Diptera than Neuroptera or Dermaptera, we will refer our results concerning the mode of follicular cells diversification in *Pieris* mostly to *Drosophila*.

In the fruit fly, the egg chamber is built of the cluster of 16 germline cells surrounded by follicular epithelium. The following follicular cell subpopulations have been described: anterior and posterior polar cells, border cells, main body cells, centripetal cells, stretched cells, posterior terminal cells, dorso-lateral cells, and interfollicular stalk cells. The first to be specified within the epithelium are two pairs of follicular cells located at the opposite extremities of the egg chamber (polar cells) and the interfollicular stalk cells that directly contact the polar cells (Deng and Bownes 1998; Grammont and Irvine 2002; Assa-Kunik et al. 2007; Wu et al. 2008). *Drosophila* anterior polar cells are responsible for patterning of the surrounding follicular epithelium into three subpopulations: border, stretched, and centripetal cells (Deng and Bownes 1998; Grammont and Irvine 2002; Torres et al. 2003; Assa-Kunik et al. 2007).

In *Pieris*, the cluster counts eight germline cells. Both in *Pieris* and *Drosophila*, like in many other insects with polytrophic ovaries (Büning 1994), the formation of the germ cell clusters results from incomplete and synchronous divisions of cystocytes, and the number of germ cells (*N*) is in accordance with Giardina’s rule (*N* = 2^*n*^, where “n” is the number of divisions) (Giardina 1901). Previous studies on nematoceran flies have shown that in *Tinearia* (16-cell germ clusters) and *Chaoborus* (8-cell germ clusters), the general pattern of follicular cells’ diversification is similar (Mazurkiewicz-Kania et al. 2012). Therefore, it seems reasonable to assume that in *Pieris*, in comparison to *Drosophila*, distinct mode of follicular cells’ diversification does not result from different number of the germline cells. In Pieris, the sequence of appearance of the follicular cell subpopulations is distinct. The first subpopulations that differentiate are the mbFC and stFC (Fig. 5). Polar cells sensu *Drosophila* do not exist. Polar cells have been identified neither in Neuroptera nor in Dermaptera (Tworzydlo et al. 2005; Garbiec and Kubrakiewicz 2012). Such follicular cells have been distinguished only on the basis of morphological studies in other (than *Drosophila*) higher and lower dipterans (Kubrakiewicz et al. 2003; Mazurkiewicz and Kubrakiewicz 2005, 2008; Tworzydlo et al. 2005; Jaglarz et al. 2008, 2009, 2010; Mazurkiewicz-Kania et al. 2012). In *Pieris*, posterior terminal cells are positioned at the posterior pole of the oocyte, and similarly to *Drosophila*, they do not directly contact the basal lamina. Since in *Pieris*, a molecular identity of posterior terminal cells is unknown; they should be considered only as equivalents of *Drosophila* posterior polar cells. Posterior terminal follicular cells in *Pieris* originate from mainbody follicular cells as the third subpopulation.

In *Pieris*, differentiation of centripetal cells happens much earlier compared to the fruit fly (Deng and Bownes 1998; Grammont and Irvine 2002; Denef and Schüpbach 2003; Horne-Badovinac and Bilder 2005; Ogienko et al. 2007, 2013; Wu et al. 2008). However, in *Drosophila*, these cells secrete the chorion of the anterior pole of the egg capsule. Moreover, in *Pieris*, centripetal cells are engaged in micropyle formation. In contrast, in all investigated lower and higher dipterans (Nematocera and Brachycera), the micropyle apparatus is produced by the highly specialized follicular cells of the anterior pole of the egg chamber (in *Drosophila* defined as the border cells) (Kubrakiewicz et al. 2003; Mazurkiewicz and Kubrakiewicz 2005, 2008; Tworzydlo et al. 2005; Jaglarz et al. 2008, 2009, 2010; Mazurkiewicz-Kania et al. 2012). In many nondipteran insects with the polytrophic type of ovary (including butterflies) (Zelazowska 2005; Tworzydlo and Kisiel 2011; Garbiec and Kubrakiewicz 2012; Garbiec et al. 2016; and the present study), these specialized cells do not exist.

In *Drosophila*, the interfollicular stalk cells differentiate at the very beginning of follicular epithelium morphogenesis and together with the polar cells, they cooperate in egg chamber polarization (Grammont and Irvine 2002; Torres et al. 2003; Roth and Lynch 2009). In *Pieris*, the interfollicular stalk cells develop from the mainbody follicular cells at the final stage of the follicular cells’ differentiation. The late appearance of the interfollicular stalk cells in *Pieris* suggests that they are not involved in the establishment of the anterior-posterior axis of the oocyte/egg chamber, and their role is basically structural (joining/separating the egg chambers).

### Follicular cell migration

#### Active migration

In the egg chambers of *Drosophila* and other true flies (Brachycera), some subpopulations of follicular cells undergo directed migrations before they reach their final location within the developing egg chamber. Some follicular cells only change their relative position within the epithelial layer, while some others (e.g., the border cells) become invasive and having detached from the epithelium actively migrate within the germline compartment (Niewiadomska et al. 1999; Geisbrecht and Montell 2002; Starz-Gaiano and Montell 2004; McDonald et al. 2006; Montell 2006; Prasad and Montell 2007). In *Drosophila,* during vitellogenesis, border cells delaminate from the anterior pole of the egg chamber, form a cluster encompassing the pair of the anterior polar cells, and begin to migrate among the nurse cells toward the oocyte (Geisbrecht and Montell 2002; Ghiglione 2002; Sokol and Cooley 2003; Yoshida et al. 2004). In *Drosophila*, active migration of follicular cells also concerns centripetal cells. The latter migrate between the nurse cells and the oocyte until they come to the border cells. Among insects, active migration of border cells is characteristic only of brachycerans (Jaglarz et al. 2008, 2010). On the other hand, active migration of centripetal cells has been demonstrated in higher Brachycera (Cyclorrhapha) and other insect taxa of different phylogenetic positions such as Dermaptera (Tworzydlo and Kisiel 2011) and Lepidoptera. Within the latter group, migration of centripetal cells has been reported in *Bombyx mori* (Yamauchi and Yoshitake 1984a, 1984b), *Hyalophora cercopia*

(King and Aggarwal 1972), *Ephestia kuehniella* (Cruickshank 1973), and *Calpodes ethlius* (Griffith and Lai-Fook 1986)*.* The aforementioned data, the obtained results, and unpublished data (M. M-K) indicate that the active migration of centripetal cells in lepidopterans is common. In *Euborellia fulviceps* (Dermaptera), centripetal cells move at later stages of oogenesis to cover the anterior pole of the oocyte after dumping the nurse cell cytoplasm into the oocyte (Tworzydlo and Kisiel 2011). In *Pieris*, compared to *Drosophila* and *Euborellia*, movement of centripetal cells takes place much earlier (in previtellogenic stages), indicating that the timing of centripetal cell migration differs among insects, but the reasons for this diversity remain unknown.

#### Planar migration

In some insects, e.g., lower Diptera (Nematocera) (Mazurkiewicz and Kubrakiewicz 2005, 2008) and Neuroptera (Garbiec and Kubrakiewicz 2012; Garbiec et al. 2016), none of the follicular cell subgroups display ability to active migration. In Nematocera, some of the follicular cells (micropyle forming cells) alter their position within the egg chambers solely by planar migration (dislocations within the epithelium). Their ultimate position is the result of cell shape changes and short translocation within the epithelium (Mazurkiewicz and Kubrakiewicz 2005, 2008). One example of planar migrations is circumferential migration of the follicular cells in *Drosophila melanogaster*. In this case, the entire follicular epithelium migrates over its immobile basement membrane (Isabella and Horne-Badovinac 2015; Squarr et al. 2016; Chen et al. 2017; Duhart et al. 2017).

Intercalation is another type of planar migration that requires changes in adhesion and cytoskeletal rearrangement (Keller 2006; Shindo 2018). This type of cell movement is widespread in animal development. Cell intercalation can occur early in development, during gastrulation when the germ layers are formed, or later during organogenesis when tissues require elongation (Walck-Shannon and Hardin 2014; Shindo 2018). Diverse mechanisms of cell intercalation have been described: the crawling mode, contraction mode, and rosette formation (Shindo 2018). In the crawling mode, the actin cytoskeleton plays a key role. The cells elongate and crawl and pull the neighboring cells by actin-rich protrusions (Keller 1984; Shindo 2018). The contraction mode and rosette formation are based on remodeling of cell junctions and actomyosin accumulation (Shindo 2018). In *Pieris*, the projections of intercalating cells are rich in microfilaments. In the light of these observations, we postulate that in *P. napi*, intercalation proceeds in a way comparable to the crawling mode. To the best of our knowledge, the participation of intercalation in follicular epithelium morphogenesis in insect ovaries has been previously described only in *Drosophila*. In the fruit fly, intercalation is involved in genesis of the interfollicular stalks that occurs at the beginning of follicular epithelium differentiation (Godt and Laski 1995; Keller 2006).

The obtained results strongly indicate that construction of interfollicular stalks in *Pieris* proceeds in a different way comparing to *Drosophila*. First, the interfollicular stalks are formed much later, during advanced vitellogenic stages. Second, intercalation of mainbody follicular cells leads to significant extension of epithelium that covers the oocyte surface. Some of the “supernumerary” cells lose contact with the oocyte and slide down at its posterior pole, thus forming the interfollicular stalks. Third, in *Pieris*, the interfollicular stalk cells originate from the mbFC.

### Follicular cells in vitellogenesis and choriogenesis

Vitellogenesis is the process whereby the required yolk nutrients are deposited into growing oocytes. In many insects, yolk precursors are produced outside the ovary, e.g., in a fat body, and transported to the ooplasm by endocytosis. This process is made possible by patency of the follicular epithelium (Raikhel and Dhadialla 1992; Swevers et al. 2005). Patency of follicular cells is hormonally regulated and, in many cases, correlated with the commencement of vitellogenesis (Telfer et al. 1982; Swevers et al. 2005). In *Pieris*, spaces between follicular cells have been observed only in advanced vitellogenesis. Moreover, at the border between the oocyte and mbFC, numerous microvilli are visible, and the cytoplasm of mbFC is rich in organelles of a secretory pathway. It means that in *Pieris*, follicular cells probably participate in the synthesis of yolk precursors, like in other butterflies (Bast and Telfer 1976; Bean et al. 1988; Raikhel and Dhadialla 1992) and dipterans (Huebner et al. 1975; Geysen et al. 1987).

## Conclusions

The results presented in this paper show that the processes of differentiation and diversification of follicular epithelium in the polytrophic ovaries of *Pieris napi* are significantly different from the model scheme described in *Drosophila*. Differences concern not only the number of morphologically distinguished subpopulations of follicular cells but also highlight some of their putative function during oogenesis. Lepidoptera represents the superorder Amphiesmenoptera, which is closely related to Antliophora, including the model organism *Drosophila melanogaster*. Therefore, comparative studies of polytrophic ovaries in non-dipteran insects can shed light on determining the evolution of follicular epithelium morphogenesis in the fruit fly.

**Fig. 6:**
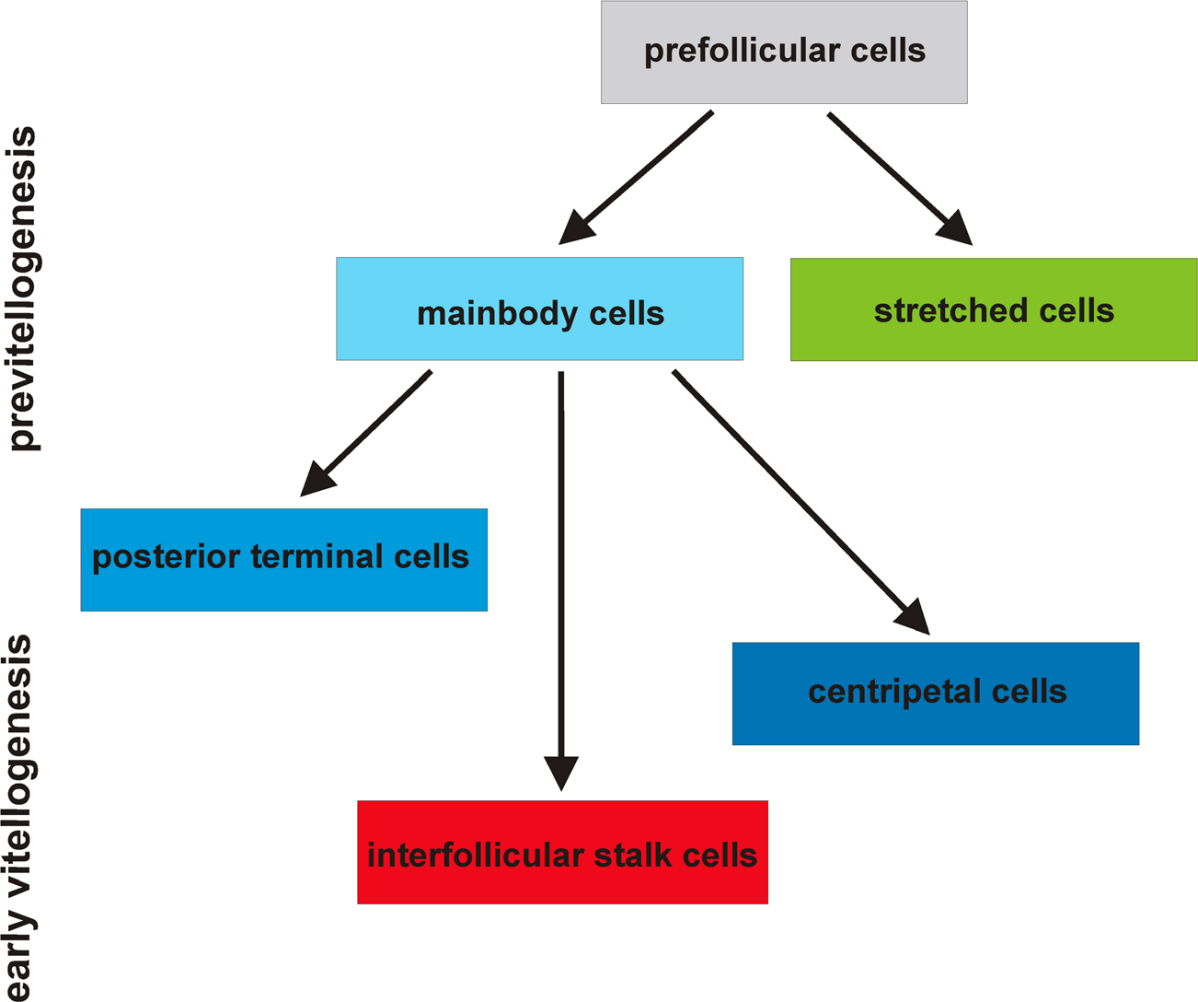
Diagram of diversification of follicular epithelium in *Pieris napi*

**Fig. 7:**
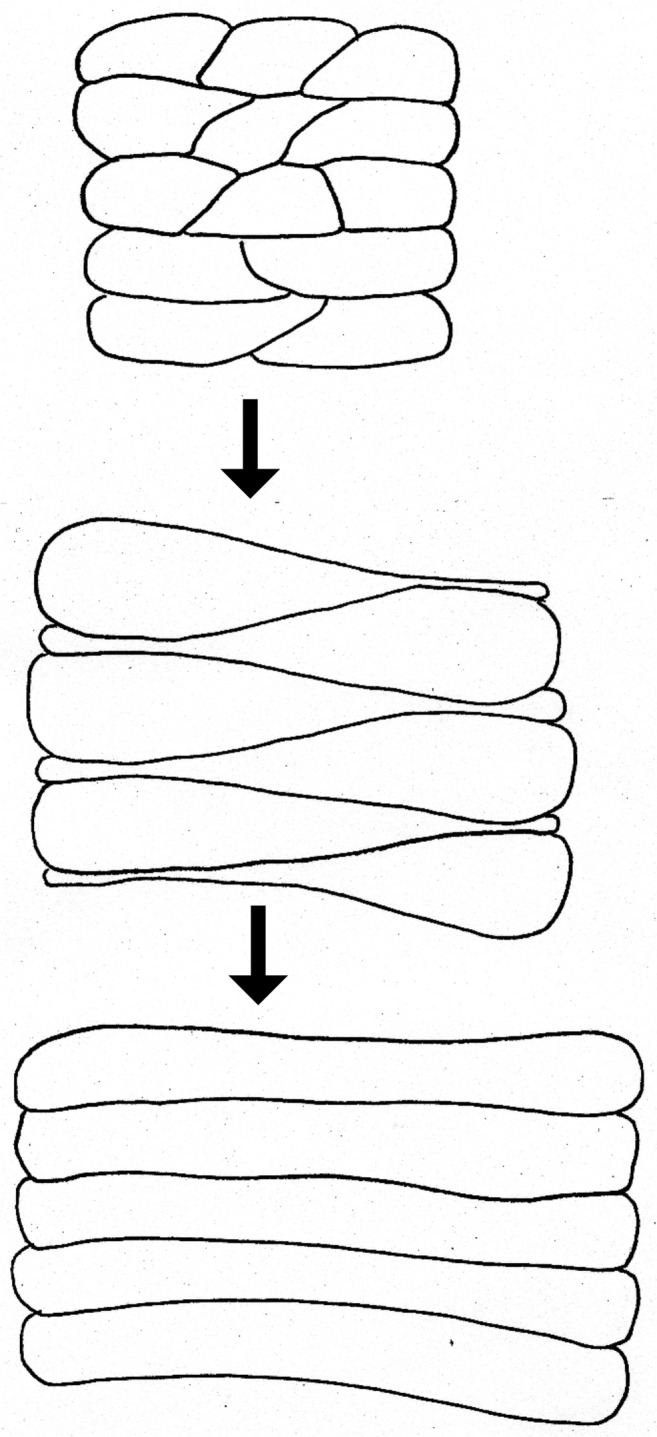
Diagram of main body follicular cell intercalation during previtellogenic/vitellogenic growth of the oocyte
